# Notes and News

**Published:** 1901-07-06

**Authors:** 


### NOTES AND NEWS.

THE Prince of Wales's Hospital Fund has received a legacy of £500 through the will of the late Miss Georgina Ellis, of Clapham Park.

THE amount received from the Churches up to the present time by the Metropolitan Hospital Sunday Fund has reached upwards of £23,000.

THE Government of New South Wales contributed £86,000 to the hospitals during the past year. The Sydney Hospital Saturday Fund Collection this year yielded £4,750.

THE number of smallpox cases in Paris is still very pronounced. The last return of deaths from the disease exceeded the average number for the period during the past nineteen years.

MR. WILLIAM QUARRIER announces that the same generous friend who anonymously gave £10,000 to erect a second Consumption Sanatorium has just increased his gift by upwards of £4,000, to furnish and complete the building and install the electric light.

ON July 10th, at 3 o'clock, Sir Henry Roscoe will open the new pathological buildings at the London Hospital and will also distribute prizes to the medical students and nursing probationers. On July 15th, at 3 o'clock, the Lord Mayor will open the new hospital of St. John and St. Elizabeth in Grove End Road, N.W.

THE Rev. Richard Free of St. Cuthbert's Lodge, Millwall, informs us that he is collecting £100 for the purpose of extending the Finsen light treatment for lupus at the London Hospital. He will be glad to receive contributions towards his object. The present installation at the London Hospital is far too small to cope with the applications for treatment.

MR. B. N. BAKER, of the Atlantic Transport Company, acting as a citizen of the United States, has presented the hospital ship *Maine* to the British Government, in appreciation of the long protection afforded to his interests under the British flag. Appreciative thanks have been sent to Mr. Baker. The fittings of the ship had previously been presented to the British nation by the American ladies who undertook all the arrangements of the *Maine*.

THE Festival Banquet of the Royal Eye Hospital will be held on Monday, July 8th, at the Hôtel Métropole, Sir J. Blundell Maple in the chair.

THE number of plague cases in India is now diminishing, but in Hong Kong it is still very active, the cases being of a virulent nature. In Egypt the disease is much scattered and isolated and of a mild type.

PROFESSOR RUDOLPH VIRCHOW, as president of a committee, is issuing invitations to prominent members of the medical profession throughout the world to attend the unveiling of a bust to Dr. Armaner Hansen, the discoverer of the bacillus of leprosy, which is to be placed in the Lungegaards Hospital at Bergen.

THE National Hospital for Consumption is issuing an appeal towards the extension of the institution and its support. So much attention has recently been called to the proper treatment of consumption that the demands for admission at Ventnor are very great. £1,000 is required before the building can be commenced.

IN America recently a Christian scientist very rashly interfered with a case of typhoid which he took over from the patient's doctor before recovery. Four days after being subjected to the influence of Christian science, the patient died. The physician was called in to sign the death certificate, in which he stated his opinion that the patient died of neglect and the interference of the Christian scientist.

THROUGH the munificence of Sir Robert and Lady Pullar, Perthshire will shortly possess a completely equipped establishment for the treatment of consumptives on the open-air system. The building is situated on a western slope at Kinnoul Hill, near Perth. Accommodation is provided for 20 patients. The cost will be not less than £8,000. Efforts are being made to obtain subscriptions towards an endowment fund of £10,000 whereby a certain number of patients from the very poorest classes may be received free of cost.

MEDICAL morality in the United States is likely to startle the more conservative Briton. It is reported that at a meeting of the Colorado State Medical Association a member proposed a legislative enactment providing that the parents of imbecile children might be permitted to arrange their painless destruction. The *Medical Journal,* commenting on the subject, says: "The proposed law is too wishy-washy. The children should be killed whether their parents consent or not, and the latter also had better be put out of the way lest they procreate other children of feeble intellect."

ADDENBROOKE'S HOSPITAL, Cambridge, seems still to be in difficulties, partly from lack of funds, partly from lack of unity in administration. It is indeed difficult to understand how any institution can carry out any reform or even adhere to any settled policy so long as its Weekly Board, the body which is entrusted with the spending of its money, is constituted as is the Weekly Board of Addenbrooke's Hospital. The first essential for good management in any institution is that the managing body shall be responsible to governors. Such a Weekly Board as that at Addenbrooke's, at which any governors who please may attend and vote, is absolutely impossible.

